# ¿Responde la Resolución 8430 de 1993 a las necesidades actuales de la ética de la investigación en salud con seres humanos en Colombia?

**DOI:** 10.7705/biomedica.4333

**Published:** 2019-09-01

**Authors:** Julio César Mateus, María Teresa Varela, Diana María Caicedo, Nhora Lucía Arias, Cruz Deisy Jaramillo, Liliana Cristina Morales, Gloria Inés Palma

**Affiliations:** 1 Facultad de Salud, Universidad del Valle, Cali, Colombia Universidad del Valle Facultad de Salud Universidad del Valle Cali Colombia; 2 Doctorado en Salud, Escuela de Salud Pública, Facultad de Salud, Universidad del Valle, Cali, Colombia Universidad del Valle Escuela de Salud Pública Facultad de Salud Universidad del Valle Cali Colombia; 3 Grupo iDIES, Fundación Universidad del Valle, Cali, Colombia Grupo iDIES Fundación Universidad del Valle Cali Colombia; 4 Grupo de Investigación en Salud y Calidad de Vida, Departamento de Sociales, Facultad de Humanidades y Ciencias Sociales, Pontificia Universidad Javeriana, Cali, Colombia Pontificia Universidad Javeriana Departamento de Sociales Facultad de Humanidades y Ciencias Sociales Pontificia Universidad Javeriana Cali Colombia; 5 Grupo de Epidemiología y Salud Poblacional, Universidad del Valle, Cali, Colombia Universidad del Valle Grupo de Epidemiología y Salud Poblacional Universidad del Valle Cali Colombia; 6 Programa de Enfermería, Universidad Libre de Cali, Cali, Colombia Programa de Enfermería Universidad Libre de Cali Cali Colombia

**Keywords:** bioética, códigos de ética, ética en investigación, revisión ética, discusiones bioéticas, ética basada en principios., Bioethics, codes of ethics, ethics, research, ethical review, bioethical issues, principle-based ethics

## Abstract

La verificación del cumplimiento de los principios éticos en la investigación en salud legitima su ejercicio ante la sociedad y posibilita la resolución de dilemas éticos frente a nuevos intereses y métodos de investigación.

En Colombia, la Resolución 8430 de 1993 es una de las principales pautas éticas que regulan la investigación en salud. Dado que no ha sido revisada ni actualizada desde su adopción, se hace necesario valorar su vigencia y suficiencia para abordar los potenciales dilemas éticos que se plantean actualmente en la investigación en salud en el país.

En este contexto, se detallan algunos vacíos y contradicciones, así como aspectos que requieren de una revisión profunda, a partir de una concepción amplia de las áreas y los métodos de investigación en salud.

Tras discutir las principales falencias e imprecisiones, se proponen alternativas para que la Resolución responda a las necesidades actuales del país frente a la ética en investigación en salud con seres humanos.

## Abstract (en)

Verifying the compliance with the ethical principles of health research legitimizes its exercise in the eyes of the society and allows for the resolution of ethical dilemmas that emerge from new research interests and methods.

Resolution 8430 of 1993 is one of the main ethical guidelines governing health research on human beings in Colombia. Considering that the resolution has not been revised or updated since its promulgation it becomes necessary to evaluate its current validity and adequacy to address the potential ethical dilemmas in the existing country’s health research.

Some gaps, contradictions, and aspects that require a deep review are detailed in this paper from a wide conception of health research areas and methods. After discussing the main weaknesses and inaccuracies, some alternatives are proposed to adjust the resolution to the present needs in health research with human beings.

El descubrimiento de los experimentos llevados a cabo en seres humanos durante el régimen nazi y algunos incidentes ocurridos en la posguerra, como la exposición de prisioneros a condiciones extremas de baja presión atmosférica, supresión de oxígeno, hipotermia, tóxicos y quemaduras; la generación intencional de heridas o enfermedades tropicales para la posterior prueba de vacunas o medicamentos; la aplicación de dosis letales de medicamentos; los experimentos psicológicos; las pruebas radiactivas secretas, y el estudio Tuskegee sobre sífilis, entre otros ^1^^-^^4^, motivaron declaraciones mundiales mediante las cuales se estableció una serie de principios éticos frente a comportamientos socialmente inaceptables cuando se investiga con seres humanos.

Los principios éticos son criterios de decisión, esto es, guías para definir las conductas que una comunidad científica debe considerar en torno a lo que se debe hacer y lo que no en las situaciones que enfrenta en su quehacer. Estos principios se recogen en pautas, códigos o declaraciones éticas. En la mayoría de dichas pautas, los principios éticos comunes son el respeto a las personas -también denominado autonomía personal-, la beneficencia y la justicia ^4^^-^^9^.

Las pautas éticas constituyen un marco de referencia para evaluar los métodos de la investigación en seres humanos antes de implementarlos y predecir los efectos que podrían tener sobre los participantes ^4^^-^^9^. Su aplicación busca el equilibrio entre los derechos de los participantes y los beneficios de los hallazgos científicos, y no se limita a aspectos formales referentes al consentimiento informado, como podría pensarse, sino que abarca aspectos que incluyen a los investigadores, la calidad y la pertinencia del protocolo de la investigación y el uso que se propone para los resultados, entre otros ^10^. Además, se orientan a prevenir la ejecución de nuevas investigaciones que no tengan en cuenta la seguridad y la libre decisión de los participantes. En ese sentido, se exige que los responsables de las decisiones, el personal de salud que hace uso de los hallazgos de la investigación y los investigadores que la generan se ciñan a dichos principios éticos básicos, y se mantenga el equilibrio entre el rigor científico y el respeto a las personas ^11^^,^^12^.

Para verificar que las investigaciones en seres humanos tengan en cuenta los principios éticos establecidos, se requiere que los comités de ética revisen los protocolos de investigación antes y durante su implementación para constatar la calidad científica del estudio, la probidad del financiador, el nivel de riesgo que asumirán los participantes y los procedimientos que se implementarán para minimizarlos o atenderlos. Asimismo, los comités de ética deben contribuir a salvaguardar la dignidad, los derechos, la seguridad y el bienestar de los participantes de una investigación; proporcionar una evaluación independiente, competente y oportuna de la ética de los estudios propuestos, y hacer un seguimiento regular del aspecto ético de los estudios que recibieron su aval ^13^. Cuando esta verificación se hace de manera independiente, se contribuye a la legitimidad de la investigación ante la sociedad en general.

Ahora bien, dados los avances científicos, las nuevas prácticas de investigación y la creciente diversidad de los métodos, han surgido nuevos dilemas éticos al aplicar los principios establecidos que exigen determinar la seguridad, la efectividad, la eficacia, la accesibilidad y la calidad de tales innovaciones ^7^^,^^8^^,^^14^^-^^16^. Los dilemas éticos pueden entenderse como situaciones en las que se hace presente un aparente conflicto operativo entre dos principios éticos, de tal forma que el cumplimiento de uno de ellos implica la transgresión del otro ^17^. Algunos dilemas éticos se relacionan con el conflicto inherente entre la atención en salud de la población y la del individuo, la comercialización de la ciencia, el papel del médico como investigador y el del sanador, la deshumanización de la ciencia, y la salvaguarda de la confidencialidad, entre otros ^18^^,^^19^.

Muchos de los dilemas éticos surgen en el momento de verificar y evaluar el cumplimiento de los principios éticos en los protocolos de investigación biomédica, de salud pública o de investigación en los servicios de salud ^20^, así como en los de la atención individual o la intervención comunitaria. No obstante, las pautas éticas hasta ahora establecidas no resuelven todos los dilemas éticos relacionados con la investigación.

Ante este dinamismo, se ha impuesto la necesidad permanente de revisar, actualizar, investigar y especificar la aplicación de estos principios en los diferentes ámbitos y en la generación de la información científica ^7^^,^^8^^,^^15^^,^^21^^-^^24^, lo que ha impulsado la investigación y la innovación en este campo, así como la revisión y el reforzamiento de las pautas éticas, para evitar su desactualización e insuficiencia. Además, cada vez se hace más necesaria una pedagogía que facilite la aplicación práctica de dichos principios ^24^^-^^32^.

Asimismo, las orientaciones nacionales frente a la investigación deben contemplar normas claras conformes con los lineamientos éticos de protección de las personas y las comunidades vulnerables ^5^^,^^8^ que proporcionen un marco analítico para la resolución de los dilemas éticos en el proceso de investigación en humanos ^6^^,^^33^.

En Colombia, además de las pautas éticas internacionales, como la Declaración de Helsinki ^5^, las guías del Council For International Organizations of Medical Sciences (CIOMS) ^7^^-^^9^, y la Declaración de Bioética y Derechos Humanos de la Unesco ^34^, los comités de ética tienen como referencia normativa para evaluar los protocolos la Resolución 8430 de 1993, por la cual se establecen las normas científicas, técnicas y administrativas para la investigación en salud ^35^, y la Resolución 2378 de 2008, en la que se adoptan las buenas prácticas clínicas para las instituciones que hacen investigaciones con medicamentos en seres humanos ^36^. Otro referente es la Ley 1374 de 2010, que crea el Consejo Nacional de Bioética y determina su integración, funciones, organización y financiación ^37^. Si bien esta ley dispuso la creación del mencionado Consejo desde el 2010, su decreto reglamentario (Decreto 384) ^38^ solo fue sancionado en el 2017 y actualmente continúa su proceso de conformación con los integrantes elegidos. La ausencia de este Consejo ha tenido efectos en la regulación actual de la investigación con seres humanos en Colombia, especialmente la Resolución 8430 de 1993, tal como lo analizaron en profundidad Rueda, et al. ^39^.

En un esfuerzo por aportar a este tema, recientemente el Departamento Administrativo de Ciencia, Tecnología e Innovación (Colciencias) adoptó la política sobre ética de la investigación, bioética e integridad científica (Resolución 0314 de 2018), en cuya formulación participaron las partes interesadas del Sistema Nacional de Ciencia, Tecnología e Innovación ^40^. En dicha Resolución se presenta un diagnóstico de la situación en el país, se definen sus objetivos y alcance, sus principios y mecanismos de seguimiento y evaluación, y se propone una ruta para fomentar la reflexión en torno a la necesidad de poner la ciencia, la tecnología y la innovación a tono con los lineamientos éticos y las buenas prácticas científicas.

En la coyuntura actual, caracterizada por un acelerado incremento en el número de grupos de investigación en el país ^41^, por la ampliación de las áreas de investigación, la introducción de nuevos procedimientos y una mayor demanda para la evaluación de los protocolos de investigación por parte de los comités de ética de las instituciones, resulta pertinente analizar algunos vacíos de las normas que rigen la investigación en salud en Colombia, específicamente la Resolución 8430 de 1993.

Dicha Resolución incluye aspectos generales sobre administración, bioética, bioseguridad y uso de animales y participantes humanos en la investigación. Los títulos I y II, relativos a las “disposiciones generales” y “la investigación en seres humanos”, son el objeto concreto de la discusión que se propone en el presente artículo. Dado que la Resolución rige desde 1993 y no ha sido actualizada o modificada, es necesario valorar su vigencia y su suficiencia para abordar los potenciales dilemas éticos que se plantean actualmente en la investigación biomédica y de salud pública con sus correspondientes métodos cualitativos, cuantitativos o mixtos, así como en la práctica de la salud pública ^25^^,^^28^^,^^30^^,^^42^^,^^43^. Asimismo, es preciso explorar los potenciales efectos de los hallazgos de esta valoración a la luz de las pautas éticas aceptadas internacionalmente.

Para ello, deben definirse, en primer lugar, algunos conceptos inherentes al tema para, posteriormente, profundizar en el análisis de la Resolución.

## Marco de conceptos y principios éticos básicos

En esta aproximación al marco conceptual de la ética de la investigación en salud, deben considerarse, ante todo, las definiciones de ética y bioética. 

La ética se define desde la perspectiva civil y profesional como un conjunto de normas morales que rigen la conducta de la persona en cualquier ámbito de la vida, en tanto que, desde el ámbito filosófico, se la define como la disciplina que sienta los fundamentos de los valores morales ^44^. 

Por otra parte, la bioética se entiende como “el uso creativo del diálogo para formular, articular y, en lo posible, resolver los dilemas que plantean la investigación y la intervención sobre la vida, la salud y el medio ambiente” ^44^. Según la UNESCO, la bioética conlleva un análisis de las cuestiones éticas planteadas por el desarrollo de las ciencias de la vida y la tecnología, y sus aplicaciones en salud en todas sus dimensiones ^34^. Desde un punto de vista práctico, la bioética aborda los conflictos éticos que surgen en el desarrollo de las ciencias de la vida ^45^.

## Investigación en salud y bioética

Es innegable la relación existente entre la investigación en diferentes áreas, incluida la salud, y el desarrollo general, y no solo económico, de una comunidad. Desde una perspectiva bioética es preciso comprender que la investigación en salud se relaciona con el desarrollo científico y tecnológico en el campo de la atención y el cuidado de la vida y la salud de una comunidad ^46^, pero también con el desarrollo humano. El desarrollo entendido como “la ampliación de las libertades de las personas en cuanto a su capacidad y posibilidad de alcanzar y hacer efectivas metas que son valiosas para ellas” ^47^, y unido a otros valores humanos como la autonomía de la persona, la igualdad y el respeto mutuo, se relaciona con la dignidad humana, concepto este central en la bioética ^46^.

Dado el impacto de la investigación en el desarrollo, esta se convierte en un tema de interés social por su repercusión en la vida humana, así como en un instrumento y una actividad sujetos al control político. Es por esto que la ética de la investigación debe tener en cuenta que la sociedad establece y define las conductas de quienes generan y usan los resultados de la investigación, no solo según las normas internacionales, sino también según las regulaciones nacionales que adaptan las pautas éticas a la cultura de cada comunidad ^48^^-^^51^. Además, la investigación debe estar regulada por el Estado para garantizar que dentro de su territorio las actividades de investigación se ajusten a los principios éticos, incorporen los intereses sociales y protejan a los individuos y las comunidades participantes. 

La interacción de estas dimensiones sociales y políticas determina cómo se hace la investigación, los valores que deben considerarse en su desarrollo y cómo dichos valores influyen en las conductas que se esperan de los investigadores y de quienes usan la información ^52^. Es en esta interrelación que la bioética define su objeto y adquiere su sentido ^53^.

Actualmente, el interés de la bioética en la investigación no solo se centra en conocer cómo se hace la investigación y sus potenciales efectos sobre los participantes, sino también en el uso que le será dado al conocimiento que genera ^27^^,^^54^.

## Ética de la generación y uso de los hallazgos científicos

En la generación de información científica en salud, se han desarrollado métodos cuantitativos y cualitativos que pueden emplearse por separado o en combinación según los objetivos que se persigan ^12^^,^^55^. A lo largo de los años se han suscitado expectativas y controversias en torno a la concepción de la investigación y la aplicación de sus resultados en salud. Asuntos como la investigación experimental en contextos de bajos recursos o con grupos vulnerables, los estudios multinacionales, las nuevas tecnologías en salud que pueden evaluarse, los estudios epidemiológicos, sociales y de salud pública, así como la investigación aplicada, la comunitaria y aquella que emplea métodos mixtos, han suscitado nuevas reflexiones en torno al cumplimiento de los principios éticos básicos o la evaluación de los efectos del posible uso de los hallazgos ^9^^,^^21^^,^^30^^,^^42^^,^^56^. Por ello, hoy las evaluaciones de los comités institucionales de ética no se limitan a valorar los protocolos de investigación de manera genérica y uniforme, sino que tienen en cuenta los métodos utiliza dos y la distinción entre investigación individual y colectiva, es decir, si se trata de la aplicación de los hallazgos científicos en la práctica de la atención individual en salud o de su uso en contextos colectivos.

## Los principios éticos básicos

En el ámbito internacional se han adoptado diversas declaraciones sobre la integridad y la protección de los participantes en los estudios de investigación, las cuales se fundan en los principios éticos básicos universalmente aceptados por la comunidad científica ^4^^-^^8^. Estos principios, publicados inicialmente en el Informe Belmont en 1979 ^6^, fueron producto de la discusión sobre los problemas éticos de la investigación experimental llevada a cabo por la National Commission for the Protection of Human Subjects of Biomedical and Behavioral Research , e incluyen el respeto a las personas, la beneficencia y la justicia, tal como se definen a continuación.

El principio de respeto por las personas reconoce la capacidad de los individuos para sopesar la información ofrecida por los investigadores y decidir sobre la conveniencia de participar o no en una investigación. Incluye, además, el trato digno y la protección de la autonomía disminuida o deteriorada y la exigencia de hacer investigación biomédica científicamente válida y pertinente. El principio de beneficencia se refiere a la obligación ética de maximizar el beneficio y minimizar el daño, privilegiando el bienestar de los sujetos de la investigación frente a los intereses de la ciencia y la sociedad. Por último, el principio de justicia establece la distribución equitativa de riesgos y beneficios para quienes participan en una investigación ^6^.

## Aspectos críticos de la Resolución 8430 de 1993

Una vez definidos los conceptos centrales, nuestro análisis se centra en los títulos I y II de la Resolución 8430 de 1993 ^35^, relacionados con las disposiciones generales y la investigación con seres humanos, respectivamente, y en la discusión sobre si la Resolución responde a las necesidades actuales de la ética en investigación en salud con seres humanos en Colombia. Si bien la Resolución establece requisitos para la investigación en salud, se enfoca fundamentalmente en la investigación clínica de carácter experimental, y dado que esta es solo uno de los tipos de investigación en salud, los aspectos abordados son insuficientes para otro tipo de investigaciones biomédicas y de salud pública que requieren directrices acordes con sus necesidades.

En cuanto a la investigación en salud pública, en las disposiciones generales (Título I) de la Resolución debería determinarse lo que se considera una investigación en salud pública para evitar imprecisiones. En este sentido, los Centers for Disease Control and Prevention (CDC) de Estados Unidos actualizaron en el 2010 la definición de investigación en salud pública para diferenciarla de otras actividades de salud pública que no se consideran investigación ^57^. La definición señala que el propósito de la investigación en salud pública es desarrollar o contribuir a la generación de conocimiento para mejorar la práctica en salud pública, por lo tanto, dicho conocimiento debe poder aplicarse a la comunidad. Estas características diferencian la investigación en salud pública de las actividades encaminadas a la prevención, la detección y el control de un problema de salud, o a mejorar un programa de salud pública o los servicios de salud, ya que los beneficios de estas actividades son exclusivos para el participante y los datos recolectados son para evaluar o hacer seguimiento de un programa o servicio. Así, se establece que las actividades relacionadas con la vigilancia en salud pública, la atención de emergencias, el seguimiento y la evaluación de programas, no se consideran investigación, a pesar de que podrían generar estudios que contribuyan a generar nuevos conocimientos generalizables ^57^. En este sentido, es recomendable avanzar hacia una definición semejante en Colombia para orientar de forma más precisa las normas éticas de la investigación en salud pública.

Por otro lado, en el Título II se mencionan aspectos relacionados con los principios éticos; sin embargo, no se los menciona como rectores de la investigación ni se definen sus fundamentos. En el artículo 5 se enfatiza que en toda investigación deberá prevalecer el criterio del respeto a la dignidad de la persona, pero no se lo enuncia como un principio ético sino como un criterio, lo que constituye una diferencia importante, pues le da un estatus diferente. Además, se menciona la protección de los derechos y el bienestar del sujeto de estudio, pero no se hace una referencia clara y más ilustrativa de los principios de beneficencia, no maleficencia y justicia. En este sentido, sería pertinente incluir la definición de dichos principios en la Resolución para darles la importancia requerida.

Por otra parte, en el artículo 6 se establecen los criterios que deben guiar la investigación en seres humanos. En el literal f, por ejemplo, se establece que esta debe realizarse bajo la responsabilidad de una entidad de salud, directriz que restringe la investigación únicamente a los servicios de atención y es específica para los estudios en ámbitos clínicos y, por ende, coarta la posibilidad de llevar a cabo estudios en otros ámbitos como los centros académicos, que no están dedicados a la atención de personas, los contextos comunitarios y los grupos de investigación social, entre otros, lo que podría entorpecer la conformación de redes de investigación entre grupos de diferentes sectores. Por lo tanto, es necesario que las pautas éticas nacionales también reconozcan las capacidades de investigación de instituciones que no están involucradas en la atención de personas y permitan la creación de redes de investigación interdisciplinarias, pues gran parte del conocimiento indispensable para la promoción de la salud y la prevención primaria se genera por fuera de los ámbitos hospitalarios.

Por otro lado, en el artículo 11 de la Resolución se clasifican las investigaciones según el riesgo para los participantes como sin riesgo, con riesgo mínimo y con riesgo mayor que el mínimo. La investigación sin riesgo, frente a la cual cabe mencionar que ninguna otra norma o declaración internacional la plantea, se define como aquella en que no se identifica a los participantes, o en la que no se tratan aspectos sensibles de la conducta, o en la cual se aplican técnicas de revisión documental retrospectivas, revisión de historias clínicas, entrevistas, cuestionarios y otras que no entrañan ningún tipo de riesgo para los participantes. Con esta definición se asume que el uso de la revisión documental, de cuestionarios y de entrevistas nunca pondría en riesgo el anonimato y la autodeterminación de los participantes. Otra asunción importante es que si los investigadores o revisores del protocolo de investigación consideran que los cuestionarios o entrevistas no plantean temas sensibles para los participantes, ello es suficiente para afirmar que, efectivamente, tal situación no se dará. Este planteamiento desconoce la complejidad de los procesos psicológicos que pueden darse en un entrevistado en el momento de responder a las preguntas del investigador y, por lo tanto, el riesgo de perturbarlo ^58^. Así, no es infrecuente que para algunos individuos un interrogante resulte sensible y para otros no lo sea, incluso preguntas sobre la identificación o que indaguen la composición familiar, aparentemente neutras, pueden despertar sensibilidades en algunas personas o en determinados contextos, con los consecuentes riesgos psicosociales ^59^. En este sentido, es necesario tener en cuenta la clasificación de los riesgos en los lineamientos internacionales para reconsiderar la que está vigente en Colombia.

Otro aspecto que debe considerarse a la hora de determinar el riesgo al que pueden estar expuestos los participantes de una investigación en salud, es que la Resolución 8430 responsabiliza al investigador por dicha clasificación (artículo 10), lo cual puede traducirse en una evaluación deficiente del riesgo, dado el interés de los investigadores por llevar a cabo sus proyectos. En este contexto, las normas nacionales deberían tener en cuenta lo planteado en las pautas del CIOMS ^8^, en las cuales se especifica que son los comités de ética los que deben evaluar el riesgo como parte integral de la ponderación requerida para establecer el equilibrio entre este y los beneficios para los participantes.

En la evaluación del riesgo, los investigadores y los comités de ética se enfrentan a decisiones y asuntos relacionados con la inclusión o la exclusión de ciertos grupos poblacionales considerados especiales. Las poblaciones especiales son aquellas en las que convergen condiciones de vulnerabilidad, dependencia, capacidad o aptitud ^60^.

Si bien la Resolución 8430 no incorpora el concepto de vulnerabilidad, regula la investigación en algunas poblaciones especiales, como los grupos subordinados, las personas con discapacidad y los menores de edad, así como las mujeres embarazadas, el feto y los embriones, pero no incluye a los ancianos y a las mujeres en general, algo que en nuestro contexto podría ser pertinente. La vulnerabilidad es una de las situaciones en las que podrían estar las poblaciones especiales, pero no es necesariamente lo que las define ^60^. 

El término ‘vulnerabilidad’ alude a una incapacidad sustancial para proteger los propios intereses debido a impedimentos como la imposibilidad de otorgar el consentimiento informado, la falta de medios alternativos para conseguir atención médica u otros servicios de alto costo, o la pertenencia como miembro subordinado de un grupo jerárquico ^7^, lo que implica que están más expuestos a los riesgos y tienen menos capacidad para defenderse contra los abusos ^61^. Se consideran vulnerables los individuos o grupos que no pueden ejercer plenamente su autonomía, pues son relativa o absolutamente incapaces de proteger sus propios intereses y su integridad personal ^62^.

En cuanto a los grupos subordinados, la Resolución incluye a estudiantes, trabajadores de laboratorios y hospitales, empleados y miembros de las fuerzas armadas, internos en reclusorios o centros de readaptación social y otros grupos especiales de la población, en los que el consentimiento informado puede verse influenciado por alguna autoridad. Sin embargo, no contempla el hecho de que una justificación importante para estudiar este tipo de participantes es el interés específico en mejorar sus condiciones de vida, lo cual violaría el principio de beneficencia de estos grupos. En este sentido, la Resolución debería especificar los parámetros del proceso de obtención del consentimiento informado en estos casos y solicitar de forma explícita que se aclare cómo la investigación puede brindarles beneficios.

Con respecto a la discapacidad, la Resolución la asume como un todo, sin hacer distinción entre la física y la mental, ni considerar sus niveles. En este contexto, es importante tener en cuenta que el ejercicio de la autonomía de una persona con discapacidad participante en una investigación se verá afectada, en mayor o en menor medida, por el tipo y el grado de compromiso de su discapacidad, pues no todas las personas con discapacidad mental tienen dificultades para elegir, y no todas aquellas con discapacidad física están posibilitadas para hacerlo. En cuanto a la gradualidad de la discapacidad, se sabe que las personas no son completamente capaces o incapaces de elegir; además, el riesgo que se asume debe ser proporcional a la capacidad, ya que cuanto mayor sea este, mayor deberá ser la exigencia de capacidad ^63^. En consecuencia, las normas nacionales deberían contemplar, en primera instancia, si la decisión sobre la participación puede ser asumida o no por el participante mismo, o si se le delega a su representante legal. En este sentido, cuando los beneficios de la investigación son explícitos, sería necesario evaluar si una persona en situación de discapacidad tiene la capacidad suficiente para analizar y comprender los riesgos que conlleva su participación, o si dicha decisión se debe delegar en su representante legal.

Asimismo, el artículo 25 de la Resolución plantea que en los estudios con personas en condición de discapacidad, además del consentimiento informado del representante legal, deberá obtenerse la certificación de un neurólogo, un psiquiatra o un psicólogo sobre la capacidad de entendimiento, razonamiento y lógica de la persona, lo cual plantea varios aspectos problemáticos. 

En primer lugar, si bien se menciona el tipo de profesionales que pueden realizar dicha evaluación, no se establece el tipo de valoración que deben hacer, ni se define explícitamente lo que debe entenderse por cada aspecto objeto de la evaluación. De hecho, las propuestas de evaluación cognitiva reconocidas a nivel internacional señalan otro tipo de criterios. Por ejemplo, desde la perspectiva psiquiátrica se plantea la comprensión de la información sobre lo que implica participar en el estudio; el análisis de la información frente a la situación de la persona y las consecuencias de la decisión; la utilización de la información relevante para comparar las diferentes alternativas y sus consecuencias, y la comunicación adecuada de la opción elegida. Desde la perspectiva neurológica y psicológica, no deben ignorarse los sustratos biológicos de los aspectos psicológicos involucrados en la adopción de decisiones y, por lo tanto, deben evaluarse los procesos cognitivos de atención, memoria, lenguaje, percepción espacial, cálculo y comunicación, razonamiento y actividad emotiva y afectiva ^63^^-^^66^. 

Un segundo aspecto se refiere al tipo de instrumento de medida empleado en la investigación. En torno a este aspecto, hay un debate en curso sobre cómo debe evaluarse la capacidad cognitiva, para cuya medición se utilizan pruebas neuropsicológicas, así como protocolos de evaluación de la capacidad generales y específicos, cada uno con sus alcances y limitaciones; los protocolos generales son los más aceptados y desarrollados, pues incorporan sistemas de puntuación y, en algunos casos, escenarios hipotéticos de evaluación ^66^. 

En cualquier caso, independientemente de la clasificación que se use, la evaluación de estos aspectos es en extremo difícil. Además, el uso de los instrumentos más idóneos implica altos costos y una logística muy compleja, por lo que en la Resolución no se debería exigir dicha valoración de manera indiscriminada, pues en los casos en los que haya un diagnóstico legal de discapacidad o una razón clara para sospechar que el participante no puede tomar por sí solo la decisión de participar ^63^, el representante legal puede firmar. Al obviarse esta valoración, se disminuirían las exigencias financieras y logísticas de los proyectos de investigación. 

Otro aspecto de la Resolución que amerita consideración es que equipara a los menores de edad con las personas discapacitadas y se establecen las mismas disposiciones para la evaluación de la capacidad cognitiva de ambos grupos. Según Ventura, et al. ^63^, en el caso de los menores de edad no debe hablarse de capacidad sino de madurez, y dicha madurez puede variar de manera importante entre culturas. Mientras que la capacidad de decisión de una persona adulta siempre se presupone, y es su incapacidad la que debe demostrarse ^67^, en la evaluación de la capacidad cognitiva de los menores esto se invierte, de manera que lo que debe demostrarse es si el menor tiene la capacidad, es decir, si es lo suficientemente maduro para decidir sobre su participación en una investigación ^63^. 

Dado que actualmente el menor de edad se concibe como un sujeto activo, “se le reconoce progresivamente autonomía para gestionar y participar en los asuntos que le afectan” ^68^. Además, la minoría de edad no implica que los menores sean tratados como participantes incapaces, pero dado que pueden ser más vulnerables al condicionamiento de sus decisiones, en la Resolución debería incorporarse un apartado específico que se ajuste a las características especiales que demanda su participación en investigaciones con diferentes niveles de riesgo, aspecto que no se plantea, más allá de mencionar que el consentimiento informado debe ser firmado por los padres o el representante legal, y que al menor se le debe pedir su asentimiento. En la investigación con menores de edad en el país, hoy se plantea la necesidad de hacer una reflexión profunda sobre la protección de sus derechos que se refleje en las regulaciones sobre el tema.

Por otro lado, en el capítulo IV de la Resolución, se establecen las consideraciones éticas para la investigación con mujeres en edad fértil y en embarazo, consideraciones que suscitan reflexiones sobre su autonomía y el trato justo por parte de los investigadores. En primer lugar, en el artículo 29 se plantea que las mujeres en edad fértil pueden participar en estudios con un riesgo mayor que el mínimo si se certifica que no están embarazadas al inicio de la investigación o que es posible disminuir el riesgo de embarazo una vez el estudio esté en marcha, lo que es una directriz vaga por la poca información sobre la gestión de los riesgos y los beneficios de la participación, y puede limitar el acceso de esta población a los eventuales beneficios de los estudios. Históricamente, esta falta de información ha llevado a discriminar a las mujeres a la hora de participar en la investigación clínica ^69^, pues ha prevalecido el paternalismo. Con el ánimo de proteger la salud del feto, las mujeres quedan excluidas de los estudios, lo que ha impedido ampliar el conocimiento sobre la seguridad y los potenciales beneficios de las investigaciones en esta población.

En el artículo 30 de la Resolución se restringe la autonomía de la mujer para tomar la decisión de participar en una investigación, puesto que si está embarazada, en trabajo de parto, puerperio o lactancia, se establece que “se requiere obtener el consentimiento informado de la mujer y de su cónyuge o compañero...” y dicho consentimiento solo se podrá dispensar “en caso de incapacidad o imposibilidad fehaciente o manifiesta para proporcionarlo; porque el compañero no se haga cargo de la mujer o, bien, cuando exista riesgo inminente para la salud o la vida de la mujer, embrión, feto o recién nacido.” La Resolución debería hacer énfasis, en cambio, en que dicha decisión debe recaer principalmente en la mujer, una vez se le suministre la información suficiente para comprender los riesgos y beneficios a corto, mediano y largo plazo. Además, debe plantearse que es responsabilidad del investigador verificar que se respete la autonomía de la mujer, advirtiendo si la investigación conlleva riesgos para el embarazo y evaluando o previendo acciones que los disminuyan en caso de un potencial efecto adverso ^7^^-^^9^, y sin interferir en las decisiones que las participantes tomen sobre su maternidad en caso de que la investigación tenga pocos riesgos para el embarazo.

La obligación del consentimiento del cónyuge o compañero para la participación de una mujer embarazada en una investigación, se contrapone al principio de respeto a la mujer, dado que desconoce su autonomía para decidir. Si bien el padre biológico del embrión o feto es merecedor de respeto, el hecho de que se supedite la participación de una mujer a esta condición pone en cuestión su capacidad de decisión como persona. En aquellas condiciones en que la investigación esté relacionada con la salud del feto, la decisión sobre la participación en el estudio competería tanto a la madre como al padre, pero la decisión de este último no debe estar por encima de la decisión de la mujer. Además, el artículo en mención tiene connotaciones discriminatorias cuando estipula las situaciones en las cuales se dispensaría el consentimiento del cónyuge o compañero. No es aceptable que la legislación condicione la participación de la mujer embarazada a la decisión del cónyuge. Por tal motivo, la Resolución debería incorporar pautas relativas al respeto por la autonomía de la mujer.

En este mismo artículo se contempla que el consentimiento del cónyuge puede obviarse cuando hay riesgo inminente para la salud o la vida de la mujer, el embrión, el feto o el recién nacido, planteamiento que se aplicaría a los procesos de atención clínica más que a las actividades de investigación, pues en este caso el consentimiento se solicita antes de incorporar a la mujer al estudio, cuando aún no existe ningún riesgo inminente para la salud o la vida. En este contexto, es recomendable especificar si las pautas deben regir el desarrollo de las investigaciones, diferenciándolas claramente de las aplicables a los procesos de atención clínica.

Por su parte, en el artículo 31 se plantea que los estudios en mujeres embarazadas deberán ser precedidos por estudios en mujeres no embarazadas para demostrar su seguridad. Esta disposición de la Resolución asume que hay similitud entre la psicología y la fisiología de las mujeres, independientemente de si están embarazadas o no, algo que ha sido rebatido en la literatura especializada. En consecuencia, debe admitirse que es posible hacer estudios en mujeres embarazadas sin necesidad de hacerlos previamente en mujeres no embarazadas cuando lo que se investiga solo puede estudiarse en las primeras.

Como se mencionó anteriormente, las mujeres, especialmente las embarazadas, históricamente se han visto excluidas de las investigaciones con el argumento de que debe protegerse la salud materna y perinatal. Infortunadamente, ello ha resultado en un mal mayor, pues se las expone a intervenciones, en especial farmacológicas, cuyos beneficios o potenciales daños son desconocidos. Hoy se aducen varias razones por las cuales se debe incrementar la participación de mujeres embarazadas como sujeto de las investigaciones, entre ellas, la necesidad de desarrollar sistemas de detección temprana de riesgos y enfermedades del embarazo y de tratamientos eficaces para enfermedades propias del embarazo que aún producen morbimortalidad materna y perinatal, especialmente en mujeres de escasos recursos; así como del mejoramiento de la seguridad del feto; el manejo racional de la prescripción farmacológica para favorecer el embarazo y limitar la tendencia actual de precaución extrema en el uso de fármacos en las mujeres embarazadas, una mejor representatividad de los intereses de las mujeres embarazadas en el campo de la investigación, y un mayor acceso a los beneficios de la investigación ^70^. En definitiva, se requiere que el país aborde este debate y reflexione sobre cómo promover la participación de las mujeres embarazadas en la investigación y sus potenciales beneficios, manteniendo un equilibrio entre la importancia del conocimiento que se espera generar y la seguridad de madres e hijos.

Por otro lado, debe incluirse en la Resolución la clasificación de los grupos poblacionales especiales, y especificar los criterios y los procedimientos para promover evaluaciones más integrales y detalladas. La actual Resolución no contempla la participación de los adultos mayores, las personas en situación médica crítica o en coma, los enfermos terminales ni las minorías. En concordancia con los principios de justicia y de respeto, estos grupos deberían ser contemplados como sujetos de la investigación, pues los hallazgos pueden aportar a la comprensión de sus condiciones propias y a la generación de posibles beneficios a diferentes niveles. No obstante, deben aclararse aspectos como la representatividad, el consentimiento informado y el manejo de la información.

Con respecto a los tipos de estudios, si bien la Resolución no los menciona explícitamente, sí hace consideraciones relativas a la investigación cuantitativa, haciendo especial énfasis en los estudios clínicos experimentales, aunque dejando de lado los estudios observacionales ^6^ y cualitativos en salud ^71^^,^^72^, que cada vez son más frecuentes y pertinentes. 

Si bien los aspectos éticos que se plantean para la investigación en general, son también aplicables a la investigación cualitativa ^71^, hay desafíos éticos particulares que deben tenerse en cuenta dada la complejidad que los caracteriza en términos de sus problemas, sus métodos y la divulgación de sus resultados ^72^. Entre estos, cabe mencionar la forma de acceder a los individuos, los grupos y las comunidades; la manera de influir sobre estos; las consideraciones de los participantes en torno a sus conceptos y significados en ambientes naturales, y el diálogo entre investigador y participante, el cual sucede en un espacio intersubjetivo de involucramiento, entre otros.

En la Resolución tampoco se contempla la investigación experimental por fuera de los escenarios clínicos ^73^, lo cual ha dificultado la implementación de este tipo de investigaciones que brindan conocimientos básicos para el desarrollo de programas de promoción de la salud y de prevención primaria y secundaria de la enfermedad.

Para finalizar, tampoco se establece en la Resolución un organigrama con las funciones y mecanismos de implementación, y no se señalan de manera precisa las instituciones involucradas en el cumplimiento y la supervisión de estas normas. Se espera que la creación del Consejo Nacional de Bioética, hoy en curso, favorezca la consolidación de un sistema nacional que incorpore la evaluación ética de los protocolos de investigación en seres humanos, supervise la implementación de las normas, y brinde asesoría, asistencia técnica y educación continua en los territorios. Este Consejo Nacional tendría, entonces, que encargarse de evaluar periódicamente la vigencia y la suficiencia de las normas éticas, entre ellas la Resolución 8430, para proponer actualizaciones oportunas. 

## Conclusiones

La Resolución 8430, que rige actualmente la investigación en salud con seres humanos en el país, se formul ó en 1993 y no contempla aspectos éticos de la investigación surgidos en las últimas décadas a nivel internacional y en el contexto nacional en particular. Por ello, se hace necesaria una revisión y una actualización que permitan la reflexión y la incorporación de pautas para la aplicación de los principios y el manejo de dilemas éticos que ya han sido concertados en el ámbito internacional.

Con respecto a la suficiencia de la Resolución, es evidente que los temas que incorpora y las pautas que establece no cubren todo el espectro de la investigación en salud en seres humanos, y en algunos de los asuntos lo reglamentado es insuficiente. En este artículo se resaltan algunos vacíos y contradicciones, así como algunos aspectos que requieren de una revisión profunda, de manera que la legislación responda a las necesidades actuales de la ética en investigación en salud con seres humanos en Colombia. Se señala, asimismo, la necesidad de incorporar la definición de los principios éticos sobre los cuales debe fundamentarse la investigación; de ampliar su alcance para abordar todos los tipos de investigación en salud más allá de los de carácter experimental y las áreas de investigación más allá de la investigación clínica; de revisar aquello que se considera investigación sin riesgo; de hacer consideraciones sobre las poblaciones especiales y su autonomía para participar en las investigaciones, así como sobre los aspectos logísticos y los actores involucrados en la supervisión de las pautas éticas Se espera que la creación del Consejo Nacional de Bioética permita avanzar en este sentido, con el fin de aportar al ejercicio de la investigación en salud en Colombia con calidad y ética.

Las normas de un país en torno a la investigación en salud deben ser suficientes para abordar los temas éticos tradicionales y aquellos emergentes, de manera que haya un soporte amplio y suficiente para la labor de los comités de ética. Para ello, sería beneficioso contar con un espacio ampliamente participativo de interacción periódica en torno a la bioética, que incorpore al Consejo Nacional de Bioética, a representantes de los comités de ética de investigación y a diferentes sectores de la sociedad civil que, desde diversas perspectivas culturales y disciplinarias, discutan la norma nacional a la luz de los dilemas éticos detectados en la revisión de los protocolos de investigación y de las actualizaciones con las pautas éticas internacionales. Dicha deliberación contribuiría a que quienes formulan las políticas públicas puedan actualizar las resoluciones pertinentes y generar indicaciones operativas para los comités de ética de la investigación de las instituciones.
